# Epistaxis in hereditary hemorrhagic telangiectasia: an evidence based review of surgical management

**DOI:** 10.1186/s40463-016-0116-8

**Published:** 2016-01-12

**Authors:** Christopher J. Chin, Brian W. Rotenberg, Ian J. Witterick

**Affiliations:** Department of Otolaryngology-Head & Neck Surgery, University of Toronto, Room 413, Mount Sinai Hospital, 600 University Avenue, Toronto, ON M5G 1X5 Canada; Department of Otolaryngology-Head and Neck Surgery, Western University, Toronto, Canada

**Keywords:** HHT, Epistaxis, Osler-Weber-Rendu, Surgery

## Abstract

Patients with Hereditary Hemorrhagic Telangiectasia (HHT) frequently present with epistaxis. Up to 98 % of these patients will have epistaxis at some point in their life. There are multiple ways to deal with this problem, including conservative, medical and surgical options. We present a case and an update on the treatment options for HHT, with a focus on the newer and experimental techniques.

**Electronic supplementary material:**

The online version of this article (doi:10.1186/s40463-016-0116-8) contains supplementary material, which is available to authorized users.

## Case

A 37 year old male presented with recurrent epistaxis as well as multiple telangiectasias of his fingers and anterior tongue. He was previously diagnosed with Hereditary Hemorrhagic Telangiectasia (HHT) at an outside institution. An MRI of his head was completed which did not demonstrate any evidence of an intracranial arteriovascular malformation (AVM). Bubble cardiography, used to detect AVMs in the lungs, was also read as negative. As the finger lesions were most bothersome to the patient, the risks of benefits of treating them with a YAG laser were discussed and the patient elected to proceed.

Although the patient initially had a good result, he experienced recurrence of his digital telangiectasias approximately 1 year later, and he started to become more symptomatic from his nasal lesions. Treatment with YAG laser was again undertaken, with good result. As a result of his frequent episodes of epistaxis, the patient eventually developed iron-deficiency anemia, which was treated with iron infusions (as the patient was unable to tolerate oral iron supplementation). He required treatment with YAG laser for the telangiectasias on average every 2 years, with his anemia being maintained with iron infusions. His epistaxis was frequent, but remained controllable with pressure.

Unfortunately, he recently presented to the emergency room with shortness of breath and his hemoglobin was found to be 87. He also reported melena stools. Following transfusion and stabilization he was re-assessed in our outpatient Otolaryngology- Head & Neck Surgery clinic where management with septodermoplasty was entertained to address the epistaxis. Alternative therapies, such as coblation and Bevacizumab (Avastin) are also being considered. The patient is currently weighing the risks and benefits of these treatment options.

## Introduction

Hereditary Hemorrhagic Telangiectasia, also known as Osler-Weber-Rendu (OWR) syndrome, was described nearly 120 years ago [1]. In 1896 Rendu described a syndrome of recurrent epistaxis and telangiectasias that was distinct from hemophilia [2]; this was followed by reports from Osler [3] (in 1901) and Weber [4] (in 1907). It is the constellation of recurrent epistaxis, familial inheritance, AVMs and mucocutaneous telangiectasias that now bears their names. The prevalence of HHT is approximately 1 in 5000, but there is large geographic variability [5]. The highest report rates are in Afro-Caribbean residents of the Netherlands Antilles, with an incidence of approximately 1 in 1331 [6].

**Fig. 1:**
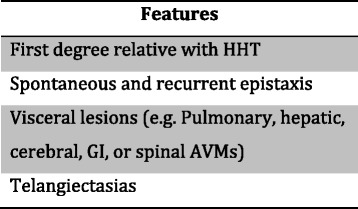
The Curaçao criteria [7]. Number of features 0–1 : unlikely, 2: possible or suspected, 3–4: definite

**Fig. 2:**
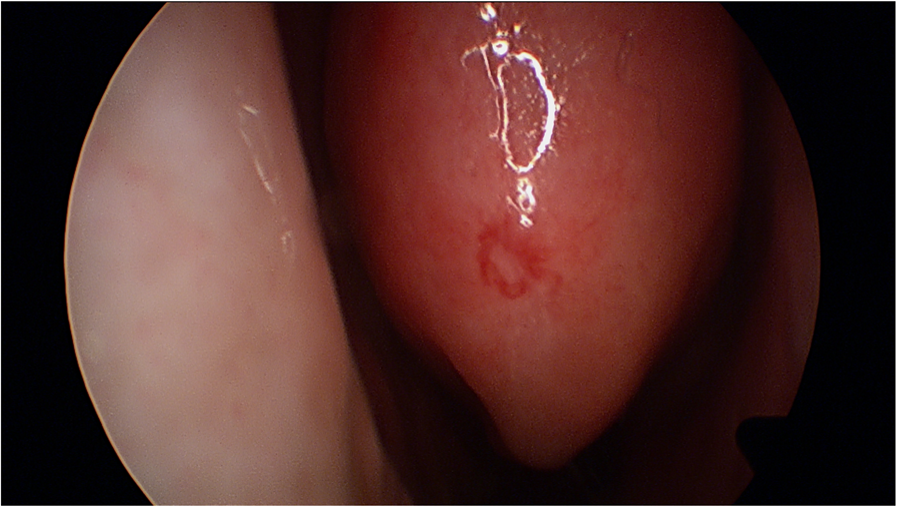
Endoscopic appearance of a telangiectasia on the head of the inferior turbinate

**Fig. 3:**
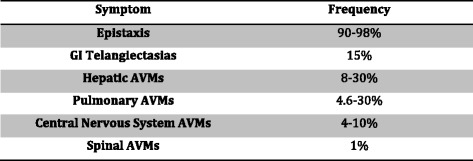
Relative frequencies of HHT manifestations [6, 9, 10]

HHT is diagnosed with the Curacao criteria (Fig. 1) [7]. Histologically, the venules are dilated and lack contractile elastic fibers, predisposing them to bleeding [1]. HHT is inherited in an autosomal dominant manner with incomplete penetrance [8, 9]. This is most often due to a defect in the ENG and ACVRL1 genes, but can be due to other genes as well, such as SMAD4 [8]. The telangiectasias that are seen are mucocutaneous in nature and have a very characteristic appearance (Fig. 2). They often appear on the lips, tongue, fingers, as well as the nasal and gastric mucosa and frequently present with bleeding (Fig. 3).

While epistaxis is the most commonly encountered symptom for Otolaryngologists, there are many other manifestations such as arterio-venous malformations (AVMs) of the lungs and gastrointestinal (GI) tract. As such, these patients are best managed in a multi-disciplinary setting [6]. International guidelines were created in 2011 that summarize the workup and management of the various manifestations of HHT [8]. In summary, treatment should be considered for asymptomatic cerebral and pulmonary AVMs because of their potential catastrophic and life-threatening presentations. When patients are symptomatic with GI, liver or oral cavity bleeding, treatment should also be considered. Close follow-up is required for all these patients.

Epistaxis is the most common symptom reported in these patients and affects up to 98 % [6, 9, 10]. Approximately 50 % will have epistaxis by the age of 20 [9]. As such, Otolaryngologist - Head and Neck Surgeons are frequently involved in the management of these patients. These episodes can range from minimally bothersome to life-threatening. The Epistaxis Severity Score is a validated and useful outcome measure that was designed specifically for HHT and can be easily used to gauge disease severity and the effect of treatment [11].

There are various different treatment options available for epistaxis in HHT, with many new advances and innovations being developed in the past few years. In the acute setting, nasal packing with absorbable material, manual pressure, and fluid resuscitation remain the mainstays of therapy. This article will focus mostly on the new surgical advances in the long-term management of this challenging disease.

## Methods

A literature review of articles and abstracts on PubMed was completed. Search terms includes “HHT” and “Osler-Weber-Rendu”, as well as “HHT epistaxis” and “Osler-Weber-Rendu epistaxis”. Abstracts were then reviewed, and those abstracts that focused on the management and treatment of HHT were selected for inclusion.

In total, 37 articles were reviewed and summaries of the evidence were generated based on these articles. Two reviewers (CJC, BWR) reviewed the articles separately to ensure concordance.

### Conservative therapy

There is a paucity of evidence regarding conservative therapy for epistaxis in HHT, and epistaxis in general. Anecdotally, it is said that patients should keep the nose well hydrated through the use of saline rinses and with humidification [8]. Barrier creams and topical emollients are also routinely used [12]. In the case of HHT, the patient must be instructed to be particularly careful when applying the treatment (barrier or saline irrigations) as the telangiectasias are extremely fragile and have a tendency to bleed when manipulated (Additional file 1: Video 1).

Recently, the group at Johns Hopkins has investigated the application of Sesame/Rose Geranium oil (RGO) for control of epistaxis in HHT. In a 2013 study, they found a significant improvement in the Epistaxis Severity Score in patients using RGO. The authors theorize that the benefit from application of RGO is nasal hydration and formation of a durable protective layer, but note is made that further investigation is required to delineate the mechanism of action [13].

Lastly, for those patients who don’t mind the aesthetics, nasal obturators are an option for HHT [14]. Based on the principle that the epistaxis is often triggered by turbulent airflow in the nasal cavity that disrupts the telangiectasias, the obturator attempts to mimic the Young’s procedure (discussed later in this article) by completely occluding the nasal cavity. There is limited, but promising, evidence for these devices in HHT [14].

*Summary: Conservative therapies have a low risk profile. Although the evidence is lacking for these therapies, they can be considered as a non-invasive first line therapy in all HHT patients with troublesome epistaxis because the risk to the patient is minimal.*

*Level of Evidence: Level 4 (1 case series)* [13]*; level 5 (1 case report)* [14]

### Medical therapy

#### Hormonal

Traditionally, estrogen was the primary agent used. Vase performed the only randomized controlled trial (RCT) in 1981 and this failed to show a statistically significant difference between estrogen and placebo [15]. In 1982, Harrison detailed the successful treatment of HHT with systemic estrogen, although this was not a randomized trial [16]. Conversely, Tamoxifen (an anti-estrogen drug used in the treatment of breast cancer) has also been noted to be effective in the treatment of HHT [17, 18].

*Summary: The evidence for hormonal therapy in HHT is limited. Some studies have demonstrated efficacy, but there is the potential for systemic side effects.*

*Level of Evidence: Level 1b (2 RCT)* [15, 18]*; level 4 (1 case series)* [16]*; level 5 (case report)* [17]

#### Bevacizumab

One of the exciting advances within the management of HHT has been the discovery of the effectiveness of Bevacizumab (Avastin) [19, 20]. Avastin is a vascular endothelial growth factor (VEGF) inhibitor, widely used in the treatment of metastatic colorectal cancer and macular degeneration. Its use intranasally has been recently studied, and it has been shown to be effective in improving both frequency and severity of epistaxis [21–25]. When Avastin was combined with the KTP laser, it was found to reduce the frequency and severity of epistaxis when compared to the laser alone; 24 % of patients in the Avastin group reported an improvement compared to the laser alone group [22]. It can be applied topically or injected submucosally, though it should be noted that injection into the cartilaginous septum has been associated with perforation (although a direct effect could not be attributed as many of these patients also underwent some sort of other septal cautery) [21, 22].

*Summary: There is recent evidence that suggests Bevacizumab can reduce the frequency and severity of epistaxis in HHT patients. Care should be exercised when injecting into the cartilaginous septum so as to decrease risk of septal perforation.*

*Level of Evidence: Level 1b (1 RCT)* [25]*; level 3b (1 case -control study)* [22]*; level 4 (3 case series)* [21, 23, 24]*; level 5 (2 case reports)* [19, 20]

#### Alternative therapies

Timolol is a beta-blocker that has also been described in the treatment of HHT. It is applied topically to the nasal mucosa three times per day. A review of the literature demonstrates just 2 case reports about it, and note is made of the risk of bradycardia as a result of the medication [26, 27]. Caution therefore should be exercised when prescribing this medication.

*Summary: There is limited evidence to support the use Timolol, with the potential for harm (bradycardia).*

*Level of Evidence: Level 5 (2 case reports)* [26, 27]

Another potential medical therapy that may be useful in the future is Thalidomide. It is felt that Thalidomide induces vessel maturation, which helps reduce the friability of the vessels [28]. However, a recent case report described a deep vein thrombosis in a patient shortly after initiation of Thalidomide [29]. A systematic review by Franchini concluded that Thalidomide was a potential option for patients with refractory epistaxis [30]. As a result of the challenges in managing anticoagulation in this group, alternative therapies should be considered.

*Summary: There is limited evidence supporting the use Thalidomide in HHT. A recent review suggests it may be useful in refractory cases.*

*Level of Evidence: Level 3a (1 systematic review of case–control studies)* [30]*; level 5 (1 case report, 1 basic science study)* [28, 29]

Sclerotherapy with sodium tetradecyl sulfate (STS) was described for use in HHT in 2011 [31]. A follow-up prospective trial recently demonstrated the efficacy of sclerotherapy when compared to “standard therapy” (defined in this study as any treatment that the patient had previously undergone, such as packing or cautery) [32]. Sclerotherapy works via injection of STS into the vascular lesion, which theoretically leads to thickening of the vessel wall and subsequently less bleeding. This is a promising new treatment that can be considered in HHT patients.

*Summary: The existing studies show benefit for sclerotherapy. Further research into this treatment modality is warranted.*

*Level of Evidence: Level 1b (1 RCT)* [32]*; level 4 (1 case series)* [31]

Tranexamic Acid (TA) is a competitive inhibitor to plasminogen and works as an anti-fibrinolytic. Tranexamic Acid has recently been evaluated in two randomized clinical trials. First, Geisthoff found in a randomized, placebo-controlled trial that while hemoglobin levels did not differ between the placebo and the TA group, self-reported epistaxis was improved [33]. Gaillard found in their randomized, placebo-controlled trial that epistaxis duration was significantly shortened when patients were on TA as compared to placebo [34]. Neither of those trials identified any significant adverse events from the medication. TA is another promising option for refractory epistaxis in the HHT patient.

*Summary: The existing studies show benefit for TA. Further research is warranted.*

*Level of Evidence: Level 1b (2 RCTs)* [33, 34]

Lastly, it has been noted that oxygen free radicals may play a role in the development of telangiectasias in patients with HHT [35]. Based on this, de Gussem et al. studied whether N-acetylcysteine (NAC), a free oxygen radical scavenger, would decrease the severity of epistaxis [36]. They found that the severity and frequency of daytime epistaxis was improved following treatment with NAC. As with the other alternative therapies, further research is warranted.

*Summary: There is limited evidence to support the use of NAC currently.*

*Level of Evidence: Level 4 (1 case series)* [36]*; level 5 (1 basic science study)* [35]

### Surgery/procedural

There are many different surgical approaches available for the treatment of HHT. As with medical therapy, a stepwise approach should be considered.

#### Coagulation

Multiple techniques have been used to coagulate the telangiectasias that are seen. Bipolar cautery was shown to be a useful option for treatment of epistaxis in HHT by Ghaderi [37]. Laser photocoagulation is also a popular option, and many different wavelengths of laser exist. Typically, a laser with higher tissue penetration that uses hemoglobin as a chromophore, such as the Nd:YAG laser, is preferred over a laser with less surface penetration. One challenge with the laser is that not all lasers can be used with flexible fibres, and therefore reaching further posterior in the nose can be difficult. As well, the increased thermal damage to surrounding tissue is a concern, and is why some authors prefer to use Coblation or ultrasonic vibration devices.

In Ghaderi’s study with bipolar cautery, the laser was also used in 22 of the 42 patients, with no increase in perforation or synechiae [37]. A 2011 Danish study used a 910 nm laser and found that the time spent with active bleeding was improved 6.5 months after treatment [38]. Lastly, ultrasonic vibrations can be used for treatment of epistaxis as mentioned previously. A 2003 study looked at 2 patients who were treated with the Harmonic Scalpel, an ultrasonic device [39]. This study highlighted the lack of damage to the surrounding mucosa, but as long-term results were not presented, further research is warranted.

*Summary: The laser and bipolar are effective surgical treatments for epistaxis. Head to head comparison is lacking and is required to determine which is most effective. The data supporting use of an ultrasonic device in HHT is currently limited.*

*Level of Evidence: Level 3b (1 case–control study)* [37]*; level 4 (1 case series)* [38]*; level 5 (1 case report)* [39]

#### Coblation

One of the more recent advances in HHT, Coblation works via radiofrequency (RF) energy and breaks down molecular bonds at relatively low temperatures (40-70 °C) [40]. Three studies have looked at Coblation in HHT and it has been shown to be a useful alternative to other surgical treatments [40–42]. Like the Harmonic Scalpel, the purported advantages of Coblation are the decreased risk of damage to surrounding, healthy mucosa with subsequent crust formation and the lower temperature, which theoretically leads to less deep tissue thermal injury.

*Summary: In the few available studies, the Coblation device has been successful in improving epistaxis in HHT and could be considered a viable alternative to coagulation and laser.*

*Level of Evidence: Level 4 (3 case series)* [40–42]

#### Septodermoplasty

Septodermoplasty involves removing the affected anterior septal mucosa and replacing it with a full- or split-thickness skin graft, laying it on top of the remaining perichondrium [43]. The nasal floor mucosa and lateral wall mucosa can also be removed as needed [43]. It has been shown to reduce the need for multiple laser procedures by up to 57 % [44].

Disadvantages of septodermoplasty include long-term crusting, the fact that this procedure often doesn’t completely stop all epistaxis as telangiectasias can grow through the skin graft, and donor site morbidity. Advantages include that it is an effective procedure and it is able to address large amounts of affected mucosa with one procedure. It can also be combined with a septectomy in those patients who have a perforation; this technique has led to improved quality of life and decreased transfusion requirements in a group of patients with severe transfusion-dependent HHT [45].

*Summary: For refractory epistaxis in HHT patients, septodermoplasty can be considered an invasive, but useful, treatment.*

*Level of Evidence: Level 4 (3 case series)* [43–45]

#### Embolization

The evidence of embolization in HHT is scant. A 2012 study by Trojanowski found an initial success rate of 85 %, though the rate of epistaxis recurrence was 43 % in their follow-up group (follow-up ranged between 6–24 months) [46]. A study in 2007 compared 10 patients who had idiopathic epistaxis to 12 patients with HHT. Those in the HHT group required significantly more re-embolizations and additional surgeries when compared to the idiopathic group [47]. Because of the significant risks involved with endovascular embolization, such as stroke and blindness, endovascular embolization is usually not recommended in this patient population.

*Summary: Embolization is infrequently recommended for the management of epistaxis in HHT and the evidence is limited.*

*Level of Evidence: Level 3 (1 case–control study)* [47]*; level 4 (1 case series)* [46]

#### Young’s procedure

Nasal closure, also known as the Young’s procedure, was first described for atrophic rhinitis in 1967, and it was first used for HHT in 1994 [48]. This procedure involves closing the nasal vestibule, and can be done either unilaterally or bilaterally. A result of a bilateral Young’s procedure is anosmia. A 2012 study by Richer looked at 36 patients, all of whom were receiving regular iron or blood transfusions at the time of surgery. This study found that 30 of the 36 patients had complete cessation of epistaxis following the Young’s procedure, and none of them required transfusions for epistaxis in the follow-up period (mean follow-up was 34 months) [48]. While control of epistaxis is typically very good, this must be weighed with the anosmia and changes in taste that will be expected to occur following this surgery. A 2013 case report highlighted a patient who had a Young’s procedure and unfortunately developed epistaxis that was refractory to pressure. After having her airway secured, her oropharynx packed, and her internal maxillary arteries embolized (bilaterally), she eventually had her Young’s procedure reversed to enable placement of nasal packing. This report highlights a very rare, but life-threatening complication that can occur post Young’s procedure; mainly that when the nose is closed, the ability to pack the nose in a standard fashion is lost [49].

*Summary: Young’s procedure is effective, but it must be weighed against the expected changes in smell and taste as well as long-term risks to the patient. This procedure may have a role in select cases when employed as unilateral treatment.*

*Level of Evidence: Level 4 (1 case series)* [48]*; level 5 (1 case report)* [49]

## Conclusions

Epistaxis is the most frequent manifestation of HHT and it can range from mildly irritating to life threatening. In the past 5 years there have been significant gains in both the medical and surgical options available to the practicing clinician for use in this frustrating condition. Clinical treatment should start with conservative options, which have a very low risk profile and the chance for improvement in symptoms. The medical therapy with the best supporting body of research is Bevacizumab, Surgical therapies should be approached in a stepwise manner, escalating as needed to treat the patient while at the same time minimizing risk of septal perforation.

## Additional file

Additional file 1: Video 1. A telangiectasia of the inferior turbinate is brushed with a cotton pledget with resultant epistaxis. (MOV 7840 kb)

## Abbreviations

AVM: Arteriovascular Malformation
GI: Gastrointestinal
HHT: Hereditary Hemorrhagic Telangiectasia
NAC: N-Acetylcysteine
OWR: Osler-Weber-Rendu
RCT: Randomized Controlled Trial
RF: Radiofrequency
RGO: Rose Geranium Oil
STS: Sodium Tetradecyl Sulfate
TA: Tranexamic Acid
VEGF: Vascular Endothelial Growth Factor
